# Implementation research priorities for addressing the maternal health crisis in the USA: results from a modified Delphi study among researchers

**DOI:** 10.1186/s43058-023-00461-z

**Published:** 2023-07-21

**Authors:** Rebecca F. Hamm, Michelle H. Moniz, Inaya Wahid, Rachel Blankstein Breman, Jennifer A. Callaghan-Koru, Implementation Science for Maternal Health National Working Group, Megan Allyse, Ian Bennet, Debra Bingham, Kacie Blackman, Sarah Brewer, Jerry Cochran, Andreea Creanga, Shayna Cunningham, Ellen Daley, Carla DeSisto, Narges Farahi, Linda Franck, Sarah Goff, Stacey Griner, Sadia Haider, Anna Hansen, Samantha Harden, Kimberly Harper, Lisa Hofler, Sarah Horvath, Jeanette Ickovics, Jennifer Johnson, Heather Kaplan, Charlan Kroelinger, Elysia Larson, Huynh-Nhu (Mimi) Le, Henry Lee, Ann McAlearney, Danielle McCarthy, Lois McCloskey, Cristian Meghea, Emily Miller, Elizabeth (Libby) Mollard, Tiffany Moore Simas, Eydie Moses-Kolko, Gina Novick, Abigail Palmer Molina, Divya Patel, Neena Qasba, Nandini Raghuraman, Amy Romano, Melissa Rosenstein, Sangini Sheth, Melissa Simon, Sharla Smith, Sindhu Srinivas, Carolyn Sufrin, Rachel Tabak, Erika Thompson, Cheryl Vamos, Daniel Walker, Jackie Wallace, Jin Xiao, Lynn Yee, Chloe Zera, Nikki Zite

**Affiliations:** 1 Maternal and Child Health Research Center, Department of Obstetrics & Gynecology, University of Pennsylvania Perelman School of Medicine, Philadelphia, PA USA; 2 Leonard Davis Institute of Health Economics, University of Pennsylvania Perelman School of Medicine, Philadelphia, PA USA; 3 Department of Obstetrics and Gynecology, University of Michigan, Ann Arbor, MI USA; 4 Institute for Healthcare Policy and Innovation, University of Michigan, Ann Arbor, MI USA; 5 Department of Epidemiology and Biostatistics, Dornsife School of Public Health, Drexel University, Philadelphia, PA USA; 6 Department of Partnerships, Professional Education and Practice, School of Nursing, University of Maryland, Baltimore, MD USA; 7 Office of Community Health and Research, University of Arkansas for Medical Sciences, Springdale, AR USA; 8 Department of Obstetrics & Gynecology, University of Arkansas for Medical Sciences, Little Rock, AR USA; 9 Center for Implementation Research, University of Arkansas for Medical Sciences, Little Rock, AR USA

**Keywords:** Maternal health, Implementation science, Delphi method, Research priorities, Priority setting

## Abstract

**Background:**

Maternal health outcomes in the USA are far worse than in peer nations. Increasing implementation research in maternity care is critical to addressing quality gaps and unwarranted variations in care. Implementation research priorities have not yet been defined or well represented in the plans for maternal health research investments in the USA.

**Methods:**

This descriptive study used a modified Delphi method to solicit and rank research priorities at the intersection of implementation science and maternal health through two sequential web-based surveys. A purposeful, yet broad sample of researchers with relevant subject matter knowledge was identified through searches of published articles and grant databases. The surveys addressed five implementation research areas in maternal health: (1) practices to prioritize for broader implementation, (2) practices to prioritize for de-implementation, (3) research questions about implementation determinants, (4) research questions about implementation strategies, and (5) research questions about methods/measures.

**Results:**

Of 160 eligible researchers, 82 (51.2%) agreed to participate. Participants were predominantly female (90%) and White (75%). Sixty completed at least one of two surveys. The practices that participants prioritized for broader implementation were improved postpartum care, perinatal and postpartum mood disorder screening and management, and standardized management of hypertensive disorders of pregnancy. For de-implementation, practices believed to be most impactful if removed from or reduced in maternity care were cesarean delivery for low-risk patients and routine discontinuation of all psychiatric medications during pregnancy. The top methodological priorities of participants were improving the extent to which implementation science frameworks and measures address equity and developing approaches for involving patients in implementation research.

**Conclusions:**

Through a web-based Delphi exercise, we identified implementation research priorities that researchers consider to have the greatest potential to improve the quality of maternity care in the USA. This study also demonstrates the feasibility of using modified Delphi approaches to engage researchers in setting implementation research priorities within a clinical area.

**Supplementary Information:**

The online version contains supplementary material available at 10.1186/s43058-023-00461-z.

Contributions to the literature

- This paper presents an adaptation of the Delphi technique for setting implementation research priorities and describes the results of its application in the clinical area of maternal health.
- While research priority-setting exercises in implementation science have typically engaged a small set of experts, which may bias the results, this process supports broader participation and can be applied to other clinical areas.
- Within maternal health, this process identified clinical interventions, contextual determinants, implementation strategies, and methodological adaptations that participants considered a priority for study to improve outcomes in the USA.

## Background

Although the USA made tremendous gains in reducing maternal mortality during most of the twentieth century, this trend has reversed, and maternal mortality has steadily increased in recent decades [1]. Currently, the USA fares worse than most other high-income nations in maternal health outcomes [2, 3]. In 2019, there were 20.1 maternal deaths for every 100,000 live births [4] and five to ten times as many cases of severe maternal morbidity [5]. Maternal health is further marked by grave disparities in outcomes by race and geography, which persist even when controlling for factors such as education and insurance coverage [6].

Reviews of maternal morbidity and mortality cases find that 40 to 60% of these cases are potentially preventable [4, 7]. Although clinical guidelines and maternal safety bundles exist to standardize care for the most important contributors to morbidity and mortality [8], they are under-implemented in many maternity care settings in the USA. Inadequate implementation of guidelines and unwarranted variations in clinical practices are reflected in large differences in maternal outcomes between delivering hospitals [6, 9–12], such as fivefold differences in obstetric complication rates [11] and tenfold differences in cesarean delivery rates [10]. Quality improvement initiatives in some states have demonstrated that standardizing care for complications such as hemorrhage and hypertension can both improve outcomes and reduce racial and geographic disparities [13–15]. However, even in successful initiatives, roughly one-third of hospitals fail to make improvements [13, 16], and these initiatives rarely extend to outpatient and community settings, where improvements in care quality may be needed most.

Implementation science and research hold great potential to assist quality improvement efforts addressing the implementation gaps in maternity care. Implementation research seeks to contribute generalizable knowledge about the implementation of evidence-based practices into routine care [17, 18]. This evidence can inform the strategies used by QI initiatives whose goals are to achieve local improvements at the level of a healthcare facility, health system, or state [19]. Implementation research studies can identify contextual determinants that influence the underuse of evidence-based practices in maternity care [20, 21] and assess which implementation strategies are effective in specific contexts [22–24]. Initiatives to address the overuse of ineffective or potentially harmful practices can be aided by emerging evidence regarding the unique challenges involved with de-implementation [25, 26]. Although the potential benefits are clear, maternal health is lagging far behind other fields in the application of implementation science methods [27]. There is an urgent need for more investment in implementation research to address the maternal health crisis in the USA [28, 29].

One strategy for catalyzing research investments, and directing investments to areas that can generate the greatest impact, is establishing research priorities [30]. There are many approaches for establishing research priorities, ranging from unstructured expert panels to highly structured and replicable questionnaire-based methods [31–33]. A set of research priorities for improving maternal health in the USA was recently published by an expert panel convened by the National Institute of Child Health and Human Development (NICHD) [34]. While the priorities proposed by the NICHD panel included important epidemiologic and clinical effectiveness questions, they did not address implementation research [28, 34], leaving a dearth of guidance for funders and researchers. Knowledge gaps include which evidence-based practices are the most important to prioritize for implementation research, which ineffective practices should be the focus of de-implementation, and which implementation strategies are most promising for testing in maternity care settings. As the development of the methods (e.g., frameworks, measures, and study designs [35, 36]) for implementation science progresses, there is a further need to understand which methods are most appropriate to deploy or adapt for harmonized research in maternity care. To address these gaps, we undertook a structured exercise to establish implementation research priorities for improving maternal health in the USA.

## Methods

### Approach

We conducted a descriptive study, following research priority-setting best practices [31]. The study was organized by an interdisciplinary steering group of four maternal health researchers engaged in implementation research with backgrounds in obstetrics, maternal–fetal medicine, nursing, and public health. All steering group members have implementation science training and prior survey and/or qualitative research experience.

In the absence of priority-setting methods specific to implementation research, we considered existing methods reviewed in the health sciences literature [31–33]. We selected the Delphi technique for its ability to incorporate and synthesize input from a large and broad group of stakeholders. The Delphi technique is a consensus-building approach originally developed by the RAND Corporation [37] that involves two or more rounds of input from stakeholders [37, 38]. The first round is typically an open-ended idea-generating round in which participants submit their suggestions in response to a prompt [38]. During later rounds, participants are asked to rate the relative importance of the suggestions remaining from the prior round [38]. To increase the inclusiveness, rigor, and transparency of the Delphi process for prioritizing research questions, we incorporated several previously-published modifications: identifying participants with related scientific expertise through a literature search of published authors, soliciting research questions for specific areas of inquiry, defining multiple criteria for rating suggested research questions, and limiting the number of rounds to 2 [39]. This study was reviewed and determined to be exempt by the Institutional Review Board of the University of Pennsylvania School of Medicine (Protocol #844,389).

### Identification and recruitment of participants

For this initial priority-setting exercise, we sought to include a broad sample of researchers with subject matter knowledge in both maternal health and implementation research. To identify eligible researchers, we used a multi-step approach (see Additional file 1 for detailed descriptions). We first searched the National Institutes of Health (NIH) RePORTER system for US-based grants that included maternal health and implementation research keywords in the abstract and grants funded under any of the dissemination and implementation research funding opportunity announcements with a maternal health keyword in the abstract. For all relevant grants, we extracted the name of the principal investigator. Second, we searched PubMed in February 2021 for US-based articles that included both a maternal health and implementation research keyword and extracted the names of first and senior authors. Third, we used a snowball sampling approach to increase the diversity of the sample by asking early participants to recommend colleagues with relevant expertise, particularly those from underrepresented backgrounds. All identified researchers were sent an email invitation to participate in an Implementation Science for Maternal Health National Working Group in February 2021. The invitation described the expectations for working group volunteers (i.e., completing two brief surveys over a 3-month period) and included a survey that collected demographic data and assessed their perceived level of engagement with implementation research and maternal health research.

### Data collection

Two Delphi surveys were administered using the Qualtrics web-based survey platform in March and May of 2021. The first included open-ended questions that solicited research topics in five areas (Table 1): (1) evidence-based practices to prioritize for implementation, (2) practices not supported by evidence to be prioritized for de-implementation, (3) research questions regarding determinants of implementation in maternity care, (4) research questions regarding implementation strategies that should be studied in maternity care, and (5) research questions related to the development and/or adaptation of implementation science methods and measures for maternity care. All question prompts included explanations of implementation research concepts and an example response (see Additional file 2).

**Table 1:** Abbreviated open-ended prompts from Delphi survey 1

Research area	Abbreviated prompt(s)
1. Evidence-based interventions for broader implementation	• Provide up to three evidence-based practices that you believe should be prioritized for implementation in maternity care in the USA; select interventions that you believe have the greatest potential to improve maternal health outcomes if they were implemented more broadly.
2. Overused interventions for de-implementation	• Provide up to three clinical practices that you believe should be targeted for de-implementation; select interventions that you believe are not supported by current evidence and whose overuse is contributing to worse maternal health outcomes.
3. Determinants of implementation	• Provide up to two research questions related to contextual factors that you believe are the most important to study for improving implementation in maternity care
4. Implementation strategies	• Provide up to two research questions related to implementation strategies that you think are the most important to study in maternity care
5. Implementation research methods	• Provide up to two research questions related to the development or adaptation of implementation science methods that you believe are the most important to study for advancing implementation research in maternity care

The interdisciplinary steering group reviewed and consolidated the open-ended responses from the first survey into fixed-choice responses for the second survey. We omitted suggestions that were out of scope for the prompt (61.5% of exclusions) or only mentioned once (38.5% of exclusions). Submissions were most commonly judged out of scope when they were non-specific questions (e.g., “how do we get busy providers to use EBPs?,” “what are the training and capacity building needs?”). Of the 497 individual recommendations submitted across the five areas in survey #1, 340 (68%) were reflected in 87 consolidated items in survey #2 (Additional file 2). During consolidation, the team identified two distinct categories of questions regarding implementation strategies—effectiveness of discrete strategies and broader questions about selection, tailoring, and testing of strategies—and these were presented separately in survey #2.

Two rating approaches were used in survey #2. For clinical practices that were recommended for broader implementation/de-implementation, respondents selected the three practices that they expected to have the greatest impact on maternal health if more widely implemented (among 20 practices) or de-implemented (among 17 practices). For each of the three selected practices, respondents rated as “high,” “medium,” or “low” the feasibility of wide implementation/de-implementation in the USA, the likelihood that this would improve outcomes, and the likelihood that this would reduce disparities. For the research questions regarding determinants of implementation (12 options), implementation strategies to test for effectiveness (14 options), broader research questions regarding how/when to use implementation strategies in maternal health implementation (11 options), and methods/measures (14 options), respondents selected their preferred five from each group and ranked each set of selections in the order of their perceived importance for advancing implementation research in maternal health.

### Analysis

Descriptive statistics, including frequencies and percentages, were calculated for participant characteristics and the selection of practices and research question items in each section of survey #2 using Stata 15. For the clinical practices that respondents selected for implementation/de-implementation, the average ratings for each of the three criteria were calculated. Bubble charts were developed to visually display respondents’ relative ratings of each practice according to the multiple criteria. For the research topics and questions selected by participants, the average relative ranking of the item by those who selected it (from 1 to 5) was calculated. The prioritization results were initially shared with the Implementation Science for Maternal Health Interest Group through a virtual adjunct meeting of the 14th Annual Conference on the Science of Dissemination and Implementation in Health.

## Results

**Fig. 1:**
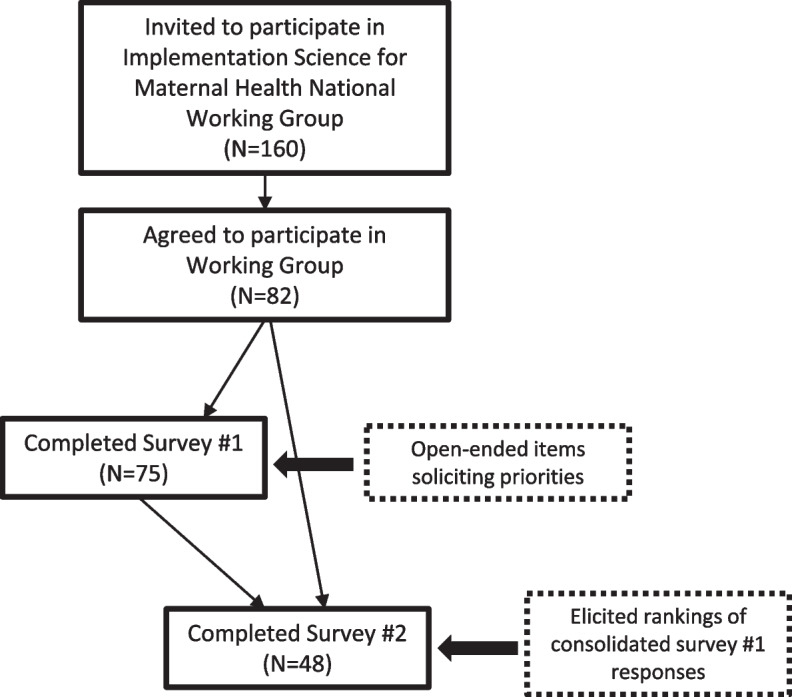
Participant flow chart

Of 160 eligible individuals, 82 (51.2%) agreed to participate in the Implementation Science for Maternal Health National Working Group (Fig. 1). Fifty-seven (69.5%) completed survey 1, which elicited open-ended responses regarding priorities. Forty-five survey 1 respondents (78.9%) and three additional working group volunteers completed survey 2, which asked participants to select and rank top choices among the consolidated responses from survey 1.

Characteristics of working group participants are detailed in Table 2. Approximately half of the participants were clinical providers, and the remaining half held other roles. Participants varied widely in the types of advanced degrees obtained and well-represented both mid- to senior and early-stage career investigators. Over 90% of participants identified as female. Nearly 75% of participants identified as White, 13% as Asian, and 7% as Black. There were no significant differences in demographic data among those who completed either survey as compared to the composition of the working group as a whole (data not shown).

**Table 2:** Characteristics of the implementation science for maternal health national working group*

	**Working group (*n* = 82), *n* (%)**
**Advanced degree(s) obtained**^a^	
MD	31 (37.8)
PhD	40 (48.8)
DrPh	3 (3.7)
MS/MHS/MSCE/MPH	32 (39.0)
MSN	6 (7.3)
Others^b^	13 (15.9)
**Role as a clinical provider in maternal healthcare**^c^	
Obstetrician	11 (13.6)
Maternal–fetal medicine physician	7 (8.6)
Midwife	3 (3.7)
Nurse	4 (4.9)
Psychologist/psychiatrist	4 (4.9)
Family medicine physician	3 (3.7)
Others^d^	10 (12.2)
None	39 (48.2)
**Location of primary appointment**	
School of Medicine	43 (52.4)
School of Public Health	15 (18.3)
School of Nursing	5 (6.1)
Other academic institutional appointment	6 (7.3)
Government agency	5 (6.1)
Others^e^	8 (9.8)
**Appointment type**^c^	
Tenured/tenure track faculty	43 (53.1)
Non-tenure track faculty	23 (28.4)
Others/not faculty	15 (18.5)
**Stage of career**	
Mid-career/senior investigator (≥ 10 years)	47 (57.3)
Early-stage investigator (< 10 years)	32 (39.0)
Trainee	3 (3.7)
**Reported high or very high level of engagement with maternal health**	59 (72.0)
**Reported high or very high level of engagement with implementation science**	46 (56.1)
**Any formal implementation science training**	45 (54.9)
**Gender identification**	
Male	8 (9.8)
Female	74 (90.2)
**Race**^**a**^	
White	62 (74.6)
Black or African-American	6 (7.3)
American Indian or Alaska Native	1 (1.2)
Asian	11 (13.4)
Others	2 (2.4)
Prefer not to answer	1 (1.2)
**Hispanic ethnicity**	2 (2.4)

^*^Data collected from February 17, 2021, to March 25, 2021

^a^ Participants were able to select more than one option

^b^ Other selections included ScD, MSW, MBA, CNM, and DDS degrees

^c^ Out of 81 responses

^d^ Other selections included dentist, endocrinologist, pediatrician, neonatologist, family planning specialist, and social worker

^e^ Other options included other clinical practice types, School of Pharmacy, and School of Social Work

Table 3 has the practices most recommended for both implementation and de-implementation in maternal health. For implementation, participants focused on (1) improved postpartum care, including home visiting programs and short interval visits; (2) perinatal and postpartum mood disorder screening and management, including collaborative care models; and (3) standardized, evidence-based practices for the management of hypertensive disorders of pregnancy. For de-implementation, practices believed to be most impactful if removed from or reduced in maternity care were (1) cesarean delivery for low-risk patients, (2) routine discontinuation of all psychiatric medications during pregnancy, and (3) routine separation of infants and parents at birth.

**Table 3:** Practices most recommended for implementation and de-implementation in survey #1, as consolidated by the investigative team

	**Number of survey #2 respondents who selected this practice in the top 3 (*n* = 48)**
**Practices most recommended for implementation**	
1. Improved postpartum care, including home visiting programs and short interval visits	20
2. Perinatal and postpartum mood disorder screening and management, including collaborative care models	14
3. Standardized, evidence-based practices for management of hypertensive disorders of pregnancy	11
4. Screening for social determinants of health as a part of prenatal care	10
5. Access to midwifery/birthing center services	10
6. Evidence-based practices for prevention of the primary cesarean, including intermittent auscultation	9
7. Telehealth as a form of prenatal/postpartum care, including remote blood pressure monitoring in pregnancy and postpartum	9
8. Contraceptive access across the lifespan, including immediate postpartum LARC	9
9. Standardized, evidence-based practices for the management of obstetric hemorrhage	7
10. Evidence-based practices for screening for and management of maternal opioid use disorder, including patient navigation services	7
11. Doula support	6
12. Implicit/racial bias training for the staff	6
13. Maternal death reporting and review committees	5
14. Group prenatal care and CenteringPregnancy	4
15. Availability of trial of labor after cesarean	3
16. Appropriate use of antenatal corticosteroids in women at risk for preterm birth	2
17. Utilization of prenatal oral health care	2
18. Low-dose aspirin for preeclampsia prevention	2
19. Nutrition and lifestyle education	2
20. Evidence-based practices for active management of labor	1
**Practices most recommended for de-implementation**	
1. Cesarean delivery for low-risk patients	23
2. Routinely discontinuing all psychiatric medications during pregnancy, without medical indication for doing so	22
3. Routine separation of infants and parents at birth	14
4. Routine continuous electronic fetal monitoring	12
5. Routine induction without medical indication	10
6. Unindicated urine drug screening during perinatal care	10
7. Excessive opioid prescribing post-cesarean	10
8. Standard 12–14 prenatal visit schedule for low-risk patients	6
9. Reduced movement in labor	6
10. Oral intake restrictions during labor	5
11. Bedrest for antenatal conditions	4
12. Unindicated ultrasounds	3
13. Maternal oxygen supplementation during labor	2
14. Overuse of vital signs in labor	2
15. Routine amniotomy	1
16. Early screening for gestational diabetes	1

**Fig. 2:**
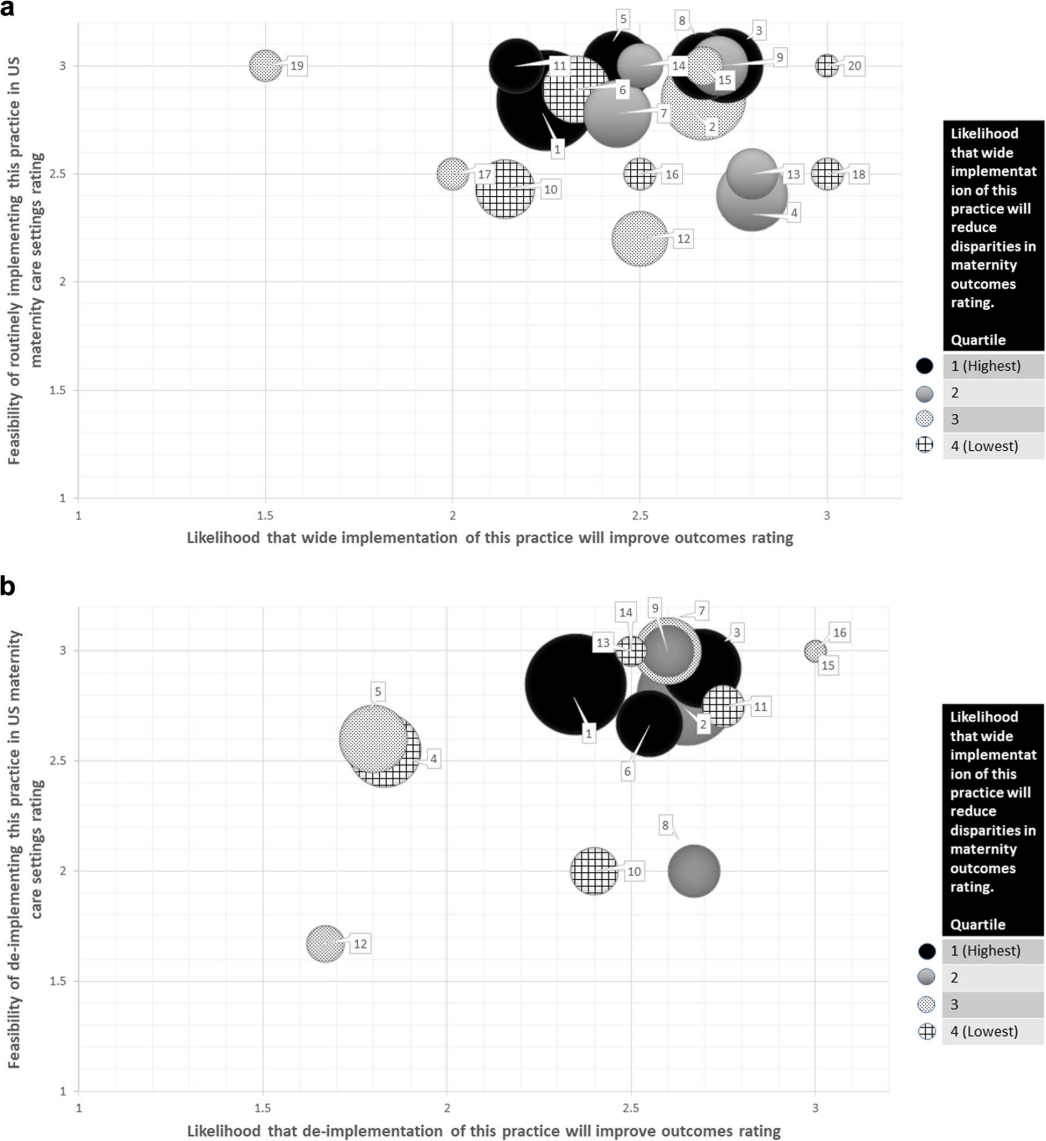
**a** Practices most recommended for implementation, as represented in a bubble chart. Numbered practices in Table 3, section A, correspond to the bubble labels. *X*-axis = feasibility of routinely implementing this practice in US maternity care settings (scale of 1–3). *Y*-axis = likelihood that wide implementation of this practice will improve outcomes (scale of 1–3). Bubble size indicates how many survey #2 participants selected the practice in their top 3 practices for implementation. Bubble color indicates the quartile of the likelihood that wide implementation of this practice will reduce disparities in maternity outcomes rating. **b** Practices most recommended for de-implementation, as represented in a bubble chart. Numbered practices in Table 3, section B, correspond to the bubble labels. *X*-axis = feasibility of de-implementing this practice in US maternity care settings (scale of 1–3). *Y*-axis = likelihood that de-implementation of this practice will improve outcomes (scale of 1–3). Bubble size indicates how many survey #2 participants selected the practice in their top 3 practices for de-implementation. Bubble color indicates the quartile of the likelihood that wide implementation of this practice will reduce disparities in maternity outcomes rating

Practices are listed in order of number of survey #2 participants who selected them to be in the top 3 practices most recommended for implementation and de-implementation. This table also serves as a legend for Figs. 1 and 2

Participants were also asked to rate their top 3 selected practices for implementation and de-implementation on the feasibility of implementation and de-implementation, likelihood of improved outcomes with implementation and de-implementation, and likely impact on reducing disparities, using a scale of 1–3 (1 = low; 3 = high). Figure 2a and b visually depict these ratings in addition to demonstrating how many participants selected the practice in their top 3 (see Additional file 3, for numeric ratings of practices recommended for implementation (Additional file 3: Table S1) and de-implementation (Additional file 3: Table S2)). While practices were generally rated highly in all domains, this depiction allows us to identify practices not only believed to be of value by many, but also believed to be feasible, with a goal of reducing disparities. For implementation, standardized, evidence-based practices for the management of hypertensive disorders of pregnancy and standardized, evidence-based practices for the management of obstetric hemorrhage come to the forefront. For de-implementation, the focus remains on routine separation of infants and parents at birth and de-implementing routine discontinuation of psychiatric medications during pregnancy.

When eliciting contextual determinants likely to exert the greatest influence on implementation in maternity care, participants focused on reimbursement policies, as well as implicit bias and racism (Table 4). In regard to implementation strategies most important to test for effectiveness in maternity care, participants selected building a coalition of partners and altering incentives to promote adoption. Three-quarters of participants selected the most important research question related to strategies to advance the field of implementation research in maternal health to be, “How can implementation strategies be selected and/or adopted specifically to promote equity?” Another commonly selected research question related to strategies was, “What implementation strategies lead to sustainability in improved implementation of evidence-based practices in maternity care?” Finally, of research goals related to methods and measures that would most help advance the field of implementation research in maternal health, participants most valued improving the extent to which implementation science frameworks and measures address social determinants of health and equity and developing approaches for involving patients in implementation and implementation research.

**Table 4:** Top ten research topics most selected to prioritize for future study in four categories

	**Number of survey #2 respondents who selected this practice in the top 5 (*n* = 48)**	**Mean ranking of priority, scales 1–5**
**Category A. Contextual determinants likely to exert the greatest influence on implementation in maternity care**		
1. Reimbursement policies	37	2.55
2. Implicit bias and racism	33	2.29
3. Unit culture (norms, values, and basic assumptions)	26	3.08
4. Organizational capacity for quality improvement/implementation	26	2.86
5. Resources of communities (e.g., internet access, transportation)	23	3.14
6. Stigma for stigmatized conditions/procedures (e.g., abortion, SUD, mental health)	19	2.84
7. The medico-legal environment	16	3.75
8. Provider workload	16	2.93
9. Provider knowledge about a clinical practice	9	3.13
10. Infrastructure of the birth setting	8	3.00
**Category B. Implementation strategies most important to test for effectiveness in maternity care**		
1. Building a coalition of partners in the implementation effort	28	2.68
2. Altering incentives to promote adoption of practices	25	2.30
3. Facilitation (e.g., guidance and interactive problem solving to support clinical practice change)	22	3.19
4. Audit provider performance and provide feedback	20	2.94
5. Perinatal quality improvement collaboratives	18	2.82
6. Preparing patients to be active participants	18	3.12
7. Accessing new funding to facilitate implementation (e.g., federal grants)	17	2.94
8. Standardized protocols	17	2.65
9. Electronic medical record changes	14	3.77
10. Digital decision support tools	13	3.18
**Category C. Research questions related to strategies that would most help advance the field of implementation research in maternal health**		
1. How can implementation strategies be selected and/or adopted specifically to promote equity?	36	2.36
2. What implementation strategies lead to sustainability in the improved implementation of evidence-based practices in maternity care?	33	2.61
3. What are best practices for engaging patients and communities in implementation work, to optimize patient-centeredness and equity?	30	2.42
4. What process should be followed to build multi-component implementation interventions (i.e., bundles of strategies) in maternity care?	24	3.23
5. How can resource-intensive implementation strategies be adapted to promote effectiveness and sustainability?	20	3.67
6. What is the effectiveness of individual implementation strategies in maternity care settings in the USA?	16	3.00
7. What is the acceptability of various implementation strategies among maternity care providers?	16	3.07
8. How can we best incentivize QI leaders to adopt an implementation science approach (e.g., measure barriers/facilitators, map to strategies, measure effectiveness)?	14	3.11
9. How does the relative effectiveness of implementation strategies vary by evidence-based practice?	13	3.38
10. What is the effectiveness of adaptive implementation strategies on the use of evidence-based practices?	12	3.44
**Category D. Research goals related to methods and measures that would most help advance the field of implementation research in maternal health**		
1. Improve the extent to which implementation science frameworks and measures address social determinants of health and equity	28	1.96
2. Develop approaches for involving patients in implementation and implementation research	27	2.87
3. Integrate implementation science methods with routine QI approaches in maternity care	24	3.09
4. Develop implementation outcomes measures that capture outcomes for both mother and baby	20	1.81
5. Generate rapid measures that reduce participant burden and increase participation among maternity care stakeholders	20	2.89
6. Develop community- and patient-level measures for determinants and outcomes of implementation	20	3.21
7. Adapt determinants frameworks to capture common determinants of implementation in maternity care settings	16	2.94
8. Develop approaches to measure and assess costs and heterogeneous reimbursements in maternity care	13	4.00
9. Incorporate common transitions of care for maternity patients in implementation frameworks and measures	11	3.22
10. Assess and model contextual moderators of implementation strategies and intervention effects	11	3.80

## Discussion

This work uses rigorous methods to establish priorities for research at the intersection of implementation science and maternal health. Specifically, this work identifies evidence-based practices most important to evaluate for implementation, as well as low-value interventions most critical to evaluate for de-implementation. Beyond evaluating specific evidence-based or low-value practices, priorities were also determined for research questions regarding determinants of implementation in maternity care, research questions regarding implementation strategies that should be studied, and research questions related to the development and/or adaptation of implementation science methods and measures for maternity care.

In 2019, NICHD convened 2 workshops to identify research gaps and priorities for maternal mortality and morbidity research in the USA [38]. Expert participants in maternal health developed consolidated lists of research gaps, challenges, and opportunities in this field. Yet, none of the findings directly addressed implementation research, a key ingredient to addressing maternal morbidity and mortality by bringing evidence-based practices to patients who need them [28]. The recent publication of NIH funding opportunities focused on implementation science within the Improving Pregnancy Outcomes Vision for Everyone (IMPROVE) Initiative [40, 41] indicates increasing recognition of the potential for implementation research to contribute to addressing the national maternal health crisis. Several recently-funded IMPROVE Initiative studies involve implementation components, including an RCT of an internet-based program for reducing perinatal depression and a sequential multiple assignment randomized trial (SMART) of a perinatal lifestyle intervention [42]. Although prior global efforts have addressed implementation research priorities for low-resource settings, this study is the first to our knowledge to specifically address priorities at the intersection of implementation science and maternal health in the USA [43–45]. Given the highly context-dependent nature of both maternal health intervention needs [46] and implementation challenges [47–49], implementation research priorities are not readily transferrable between the global setting and the USA.

Prioritizing evidence-based practices for implementation research and support is recognized as important given resource limitations [50]. In considering the clinical practices most selected for implementation focus in this exercise—improved postpartum care, perinatal mental healthcare, and standardized management of hypertensive disorders—work has only just begun. For example, improved postpartum blood pressure surveillance can occur with the implementation of innovative remote blood pressure monitoring programs [51]. In the field of perinatal mental health, implementation work is evaluating whether effective interventions can be delivered by non-specialists or even digitally [52, 53]. Implementation of nurse-driven and semiautonomous treatment algorithms for peripartum hypertension has shown promise at providing appropriately timed treatment [54, 55]. Yet, these clinical examples represent just the tip of the iceberg of implementation; larger-scale implementation studies are needed to determine how best to incorporate these practices into the diverse maternity populations and practice models that exist in the USA, including community settings where evidence-based practices are needed most.

In regard to the practices most selected for de-implementation, unnecessary cesarean delivery was the highest priority. Cesarean is considered one of the underlying causes of both maternal mortality and morbidity, and a consensus safety bundle to reduce cesarean has been the subject of several state-based implementation efforts [16, 56]. However, further researchn is needed to address de-implementation barriers, particularly related to unit culture [20]. Additionally, the perceived benefits of some obstetrics interventions are debated, including some interventions recommended by panelists for de-implementation. De-implementation decisions should take into consideration the local clinical context (e.g., staffing, resources, patient population), evidence regarding effectiveness in different contexts, and feasibility and potential unintended consequences of de-implementation.

This work also establishes the feasibility of using adapted Delphi approaches to solicit input on implementation research priorities from a broad range of researchers within a subfield. While several papers have suggested important research directions within implementation science, these have tended to focus more broadly on conceptual areas such as implementation strategies [57], sustainability [58], and mechanisms [59]. Methods that have been used or proposed for broad implementation research agenda setting include concept mapping [58], literature reviews [57], and expert panel discussions [49, 59]. A strength of the web-based Delphi approach is the ability to solicit input from a broad group of researchers to maximize the diversity of perspectives. This increases the inclusiveness of the exercises [31], as well as the reliability, which has been shown to stabilize as participation approaches 50 individuals [60]. Additionally, Delphi techniques collect and present suggestions for rating anonymously among participants to minimize bias that might result from interpersonal factors, such as deference to the most vocal or well-known participants in the group [33, 61].

Several of the Delphi modifications we incorporated were originally developed by the Child Health and Nutrition Research Initiative (CHNRI) for priority-setting exercises convened by the World Health Organization [39] and have been used repeatedly for establishing maternal and child health research priorities in low-resource contexts [32]. The survey prompts used by CHNRI [39] required adaptation to align with a focus on implementation research. The five areas in this exercise—evidence-based interventions, overused/ineffective practices, determinants of implementation, implementation strategies, and research methods—reflect major areas of inquiry in the developing field of implementation science [62]. A particular innovation of the CHNRI approach to increase rigor and transparency is defining rating criteria to make explicit the values that are being applied to rank different topics, which may otherwise be implicit and variable for different participants [31]. To lessen the survey burden and minimize between-round attrition, which can be high in Delphi exercises [33, 63], we reduced the number of items to be scored to only the participant’s top ten selections in the interventions area.

Strategies that seek input from a broad sample of researchers do present some challenges. While electronic Delphi exercises facilitate the inclusion of more participants, the format prevents discussions which can lead to rescuing of omitted suggestions, refinement of ideas, and group consensus during in-person Delphi exercises [33]. Additionally, implementation science is a relatively new field within maternal health, and the scope of implementation research questions proved difficult to conceptualize for some participants, as demonstrated by submissions that were judged to be out of scope or that closely mirrored the examples provided in question prompts. Furthermore, the inclusion of examples in question prompts may have limited the scope of responses for all participants. Similar difficulties have been observed in other research priority-setting exercises that engage broad participants [64, 65], and these difficulties were addressed by the steering committee when consolidating suggestions for the second round survey.

There was limited diversity among researchers who participated in this exercise, although the demographic characteristics of participants are likely consistent with the profile of researchers engaged in this implementation research for maternal health, highlighting the importance of efforts to increase researcher diversity. In addition, no data were collected on those who declined participation in the working group to examine if characteristics differed as compared to those who accepted participation, nor were we able to compare priority selections by participant characteristics due to the small sample size. Another major limitation of this work is the lack of inclusion of patient and community-based support individuals, such as doulas. Engaging patients in Delphi exercises to prioritize research questions may be most feasible when focused on specific clinical conditions for which patients with lived experience can be identified. Meaningful patient and community engagement may also require opportunities for discussion to clarify and resolve differences in perspective between patients and researchers [66].

## Conclusions

Increasing implementation research in maternal health has great potential to improve the quality of care and reduce poor outcomes in the USA. Research priority-setting exercises can help to generate a catalog of topics, on which there is consensus among researchers, for the field to focus on in the coming years. Such a list may help researchers direct their energies, as well as aid funders in selecting research investments. This study demonstrates the feasibility of using adapted Delphi approaches to engage researchers in setting implementation research priorities within a clinical area. Approaches for incorporating patient and community perspectives in the development of implementation research questions are also needed.

## Supplementary Information

**Additional file 1.** Search strategy.

**Additional file 2.** Survey prompts.

**Additional file 3: Table S1.** Disaggregated ratings of practices most recommended for implementation in Survey #1, as consolidated by the investigative team. **Table S2.** Disaggregated ratings of practices most recommended for de-implementation in Survey #1, as consolidated by the investigative team.

## Data Availability

De-identified datasets used for this study are available from the corresponding author upon reasonable request.

## Abbreviations

CHNRI: Child Health and Nutrition Research Initiative
IMPROVE: Improving Pregnancy Outcomes Vision for Everyone
NICHD: National Institute of Child Health and Human Development
NIH: National Institutes of Health
RePORTER: Research Portfolio Online Reporting Tools: Expenditures and Results
