# Supplementary material for: Implementation research priorities for addressing the maternal health crisis in the USA: results from a modified Delphi study among researchers
Source: Implement Sci Commun. 2023 Jul 21;4:83. doi: 10.1186/s43058-023-00461-z (PMC10360260; doi:10.1186/s43058-023-00461-z)
Supplement: Supplementary file 1 — Additional file 1. Search strategy. [file 43058_2023_461_MOESM1_ESM.docx]

**Supplemental File 1. Participant identification strategies and results**

| **Method #** | **Database** | **Strategy/ search terms** | **# results** | **# invited** | **Exclusion reasons (#)** |
| --- | --- | --- | --- | --- | --- |
| 1 | NIH Reporter: PIs | All grants funded under the Dissemination Implementation Research program announcements (PARs 02-131, 06-071, 06-520, 10-038, 10-039, 10-040, 13-054, 13-055, 13-056, 16-236, 16-237, 16-238, 18-007, 18-017, 19-274, 19-275, 19-276) with a maternal health keyword in the abstract (Maternal health keywords: Maternal OR pregnan* OR antenatal OR perinatal OR postpartum OR antepartum OR mother OR “labor and delivery” OR obstetric). | 18 | 9 | Outside the U.S. (6)  Not maternal health (3) |
| 2 | NIH Reporter: PIs | All grants with keywords for maternal health and implementation research in the abstract search using the following search string: (Maternal OR pregnan* OR antenatal OR perinatal OR postpartum OR antepartum OR mother OR “labor and delivery” OR obstetric) AND (“Implementation Science” OR “Implementation Strateg*” OR “Implementation Research” OR “Implementation study” OR “Implementation outcome*” OR “Dissemination and Implementation”). | 192 | 24 | Duplicate from search #1 (9)  Outside the U.S. (57)  Conference/network grant (12)  Consortium/center grant (44)  Other non-research (21)  Not maternal health (23)  Basic science (2) |
| 3 | Pubmed: First Authors | Keyword search with the following string: (Maternal OR pregnan* OR antenatal OR perinatal OR postpartum OR antepartum OR mother OR "labor and delivery" OR obstetric) AND ("Implementation Science" OR "Implementation Strateg*" OR "Implementation Research" OR "Implementation study" OR "Implementation outcome*" OR "Dissemination and Implementation"). | 1051 | 53 | Duplicate from search #1-2 (5)  Outside the U.S. (662)  Published prior to 2015 (208)  Not maternal health (108)  Multiple records for same author (5)  Not relevant (7)  No authors (3) |
| 4 | Pubmed: Senior Authors | Senior authors for articles identified in search #3. | 53 | 44 | Contact information could not be located (5)  Duplicate from search #1-3 (4) |
| 5 | Snowball sample | Early participants in the first survey were asked to recommend colleagues, with a focus on researchers from under-represented backgrounds. | 31 | 26 | Already identified (5) |
| 6 | Self referral | Individuals completed the working group sign up form without receiving a direct invitation. | 4 | 4 | N/A |
| **Total** | | | | **160** |  |
