# Supplementary material for: Implementation research priorities for addressing the maternal health crisis in the USA: results from a modified Delphi study among researchers
Source: Implement Sci Commun. 2023 Jul 21;4:83. doi: 10.1186/s43058-023-00461-z (PMC10360260; doi:10.1186/s43058-023-00461-z)
Supplement: Supplementary file 2 — Additional file 2. Survey prompts. [file 43058_2023_461_MOESM2_ESM.docx]

**Survey 1: Open-Ended Prompts**

**Priority Evidence-Based Practices:**The following questions ask you to provide up to three evidence-based practices that you believe should be prioritized for implementation in maternity care in the United States. Please select interventions that you believe have the greatest potential to improve maternal health outcomes if they were implemented more broadly than they currently are today.

*Example: Text-based monitoring of blood pressure.*

**Practices for De-Implementation:**The following questions ask you to provide up to three clinical practices that you believe should be targeted for de-implementation (e.g., use of the practice by healthcare practitioners and systems should be reduced or stopped). Please select practices that you believe are not supported by current evidence, and whose overuse is contributing to worse maternal health outcomes.

*Example: Continuous electronic fetal monitoring for low-risk births.*

**Determinants of Implementation:**Contextual factors can be important determinants (e.g., barriers or facilitators) for implementation of evidence-based practice in maternity care. The following questions ask you to provide up to two research questions related to contextual factors that you believe are the most important to study for improving implementation in maternity care.  You can recommend research questions related to the role of context in implementing a specific practice or in relation to general implementation efforts.

*Example: What role do reimbursement policies play in implementing long-acting reversible contraception?*

**Implementation Strategies:**Implementation strategies are interventions to promote the uptake of evidence-based practice in routine care. The following questions ask you provide up to two research questions related to implementation strategies that you think are the most important to study in maternity care.  You can recommend implementation strategies related to a specific evidence-based practice or in relation to general implementation efforts, regardless of the practice.

*Example: How effective is audit-and-feedback in changing physician practices?*

**Implementation Science Methods and Measures:**Multiple frameworks and theories have been developed to inform implementation research. The number of measures for implementation determinants and outcomes is also growing. The majority of the current frameworks, theories, and measures were originally developed in other clinical contexts and may need refinement for applications in maternity care.  The following questions ask you provide up to two research questions related to the development or adaptation of implementation science methods that you believe are the most important to study for advancing implementation research in maternity care in the United States.

Example: How should the Consolidated Framework for Implementation Research be adapted to best capture important determinants of implementation in labor and delivery units?

**Survey 2: Ranking Questions**

Part 1: Practices to prioritize for implementation

In the first survey, respondents listed evidence-based practices that they believe should be prioritized for implementation in maternity care in the United States. The consolidated list of practices recommended for implementation is provided below. **Please select up to 3 practices** that you think would have the largest impact on maternal health if they were more widely implemented. Please select practices with the greatest strength of evidence, feasibility, and likelihood of impact on maternity care outcomes and disparities (on the next few pages, you will be asked to rate the impact of the practices you selected against these criteria).

- Appropriate use of antenatal corticosteroids in women at risk for preterm birth
- Group prenatal care and CenteringPregnancy
- Utilization of prenatal oral health care
- Low-dose aspirin for preeclampsia prevention
- Nutrition and lifestyle education
- Screening for social determinants of health as a part of prenatal care
- Doula support
- Access to midwifery/birthing center services
- Evidence-based practices for prevention of the primary cesarean, including intermittent auscultation
- Availability of trial of labor after cesarean
- Standardized, evidence-based practices for management of hypertensive disorders of pregnancy
- Standardized, evidence-based practices for management of obstetric hemorrhage
- Evidence-based practices for active management of labor
- Improved postpartum care, including home visiting programs and short interval visits
- Evidence-based practices for screening for and management of maternal opioid use disorder, including patient navigation services
- Telehealth as a form of prenatal/postpartum care, including remote blood pressure monitoring in pregnancy and postpartum
- Perinatal and postpartum mood disorder screening and management, including collaborative care models
- Implicit/racial bias training for staff
- Maternal death reporting and review committees
- Contraceptive access across the lifespan, including immediate postpartum LARC

For each selected practice, please rate the evidence, feasibility, and potential impact of this practice according to the criteria below.

| Feasibility of routinely implementing this practice in US maternity care | - High - Medium - Low - I’m not sure |
| --- | --- |
| Likelihood that wide implementation of this practice will improve outcomes | - High - Medium - Low - I’m not sure |
| Likelihood that wide implementation of this practice will reduce disparities in maternity outcomes (e.g., by race-ethnicity, income, rural-urban status) | - High - Medium - Low - I’m not sure |

Part 2: Practices to prioritize for de-implementation

In the first survey, respondents also listed clinical practices that they believe should be prioritized for **de-implementation** in maternity care in the United States. The consolidated list of practices recommended for de-implementation is provided below. **Please select up to three practices** for which you think their de-implementation would have the largest impact on maternal health. Please select practices with the greatest strength of evidence for de-implementation, feasibility of de-implementing, and likelihood of impact on maternity care outcomes and disparities through de-implementation (on the next few pages, you will be asked to rate the impact of the practices you selected against these criteria).

- Routine continuous electronic fetal monitoring
- Cesarean delivery for low-risk patients
- Routine induction without medical indication
- Unindicated urine drug screening during perinatal care
- Standard 12-14 prenatal visit schedule for low-risk people
- Unindicated ultrasounds
- Maternal oxygen supplementation during labor
- Routine separation of infants and parents at birth
- Routine amniotomy
- Oral intake restrictions during labor
- Early screening for gestational diabetes
- Reduced movement in labor
- Routinely discontinuing all psychiatric medications during pregnancy, without medical indication for doing so
- Bedrest for antenatal conditions
- Overuse of vital signs in labor
- Excessive opioid prescribing post-cesarean

For each selected practice, please rate the evidence, feasibility, and potential impact of de-implementing this practice according to the criteria below.

| Feasibility of de-implementing this practice in US maternity care settings | - High - Medium - Low - I’m not sure |
| --- | --- |
| Likelihood that de-implementation of this practice will improve outcomes | - High - Medium - Low - I’m not sure |
| Likelihood that de-implementation of this practice will reduce disparities in maternity outcomes (e.g., by race-ethnicity, income, rural-urban status) | - High - Medium - Low - I’m not sure |

Part 3: Determinants of implementation in U.S. maternity care settings

In the first survey, respondents were asked to recommend research questions related to contextual determinants of implementation. The contextual determinants that were most frequently recommended for study in the first survey are consolidated in the list below. **Please select up to five** determinants that you would prioritize for research because they are likely to exert the greatest influence on implementation in maternity care. On the next page, you will be asked to rank the selected determinants in order of their importance for implementation of clinical practices.

- Reimbursement policies
- Stigma for stigmatized conditions/procedures (e.g., abortion, SUD, mental health)
- Provider specialty (e.g., OB, MFM, CNM)
- Unit culture (norms, values, and basic assumptions)
- Organizational capacity for quality improvement/implementation
- The medico-legal environment
- Provider workload
- Implicit bias and racism
- Resources of communities (e.g., internet access, transportation)
- Infrastructure of the birth setting
- Provider knowledge about a clinical practice
- Patient perceptions of a clinical practice

Part 4: Promising implementation strategies for maternity care settings

In the first survey, respondents were asked to recommend research questions related to implementation strategies. Many recommended questions addressed the effectiveness of specific implementation strategies, and those strategies are consolidated in the list below. **Please select up to five**implementation strategies that you think are most important to test for effectiveness in maternity care. On the next page, you will be asked to rank the selected implementation strategies in order of their importance for testing.

- Education and training activities for providers
- Altering incentives to promote adoption of practices
- Building a coalition of partners in the implementation effort
- Audit provider performance &amp; provide feedback
- Reminder systems for clinicians
- Facilitation (e.g., guidance &amp; interactive problem solving to support clinical practice change)
- Perinatal quality improvement collaboratives
- Electronic medical record changes
- Shift or revise roles among professionals who provide care
- Digital decision support tools
- Accessing new funding to facilitate implementation (e.g., federal grants)
- Standardized protocols
- Preparing patients to be active participants
- Clinical champion-based strategies

Part 5: Research questions related to implementation strategies

During the first survey, respondents recommended research questions related to implementation strategies. The recommended research questions are consolidated in the list below. **Please select up to five**research questions that you believe would most help advance the field of implementation research in maternal health. On the next page, you will be asked to rank the selected research questions in order of their importance for informing implementation of evidence-based practices and implementation research in maternity care.

- What is the effectiveness of individual implementation strategies in maternity care settings in the United States?
- What is the acceptability of various implementation strategies among maternity care providers?
- How does the relative effectiveness of implementation strategies differ between inpatient vs. outpatient maternity settings?
- How does the relative effectiveness of implementation strategies vary by evidence-based practice?
- What implementation strategies lead to sustainability in improved implementation of evidence-based practices in maternity care?
- What process should be followed to build multi-component implementation interventions (i.e., bundles of strategies) in maternity care?
- How can resource-intensive implementation strategies be adapted to promote effectiveness and sustainability?
- How can implementation strategies be selected and/or adopted specifically to promote equity?
- What are best practices for engaging patients and communities in implementation work, to optimize patient-centeredness and equity?
- How can we best incentivize QI leaders to adopt an implementation science approach (e.g., measure barriers/facilitators, map to strategies, measure effectiveness)?
- What is the effectiveness of adaptive implementation strategies on the use of evidence-based practices?

Part 6: Research goals related to methods & measures

During the first survey, respondents suggested research goals related to methods and measures that would be of importance to implementation research in maternity care. The recommended methodological advances are consolidated in the list below. **Please select up to five**research goals that you believe would most help advance the field of implementation research in maternal health. On the next page, you will be asked to rank the selected research goals in order of their importance for informing implementation and implementation research in maternity care.

- Develop implementation outcomes measures that capture outcomes for both mother and baby
- Adapt determinants frameworks to capture common determinants of implementation in maternity care settings
- Incorporate common transitions of care for maternity patients in implementation frameworks and measures
- Improve the extent to which implementation science frameworks and measures address social determinants of health and equity
- Generate rapid measures that reduce participant burden and increase participation among maternity care stakeholders
- Integrate implementation science methods with routine QI approaches in maternity care
- Assess the comparative utility of different implementation science frameworks when used in maternity care
- Develop community- and patient-level measures for determinants and outcomes of implementation
- Develop approaches to measure and assess costs and heterogenous reimbursements in maternity care
- Develop effective approaches for using implementation science frameworks to solicit stakeholder input for designing implementation strategies
- Develop measures of implementation readiness on labor & delivery units
- Assess and model contextual moderators of implementation strategies and intervention effects
- Incorporate systems science into implementation research in maternity care
- Develop approaches for involving patients in implementation and implementation research
