# Supplementary material for: Implementation research priorities for addressing the maternal health crisis in the USA: results from a modified Delphi study among researchers
Source: Implement Sci Commun. 2023 Jul 21;4:83. doi: 10.1186/s43058-023-00461-z (PMC10360260; doi:10.1186/s43058-023-00461-z)
Supplement: Supplementary file 3 — Additional file 3: Table S1. Disaggregated ratings of practices most recommended for implementation in Survey #1, as consolidated by the investigative team. Table S2. Disaggregated ratings of practices most recommended for de-implementation in Survey #1, as consolidated by the investigative team. [file 43058_2023_461_MOESM3_ESM.docx]

**Supplemental Table 1:** Disaggregated ratings of practices most recommended for implementation in Survey #1, as consolidated by the investigative team.

|  | **Number of survey #2 respondents who selected this practice in top 3**  **(n=48)** | **Feasibility of routinely implementing this practice in US maternity care settings**  **Scale 1-3** | **Likelihood that wide implementation of this practice will improve outcomes**  **Scale 1-3** | **Likelihood that wide implementation of this practice will reduce disparities in maternity outcomes**  **Scale 1-3** |
| --- | --- | --- | --- | --- |
| 1. Improved postpartum care, including home visiting programs and short interval visits | 20 | 2.25 (0.72) | 2.84 (0.37) | 2.94 (0.25) |
| 2. Perinatal and postpartum mood disorder screening and management, including collaborative care models | 14 | 2.67 (0.49) | 2.85 (0.38) | 2.67 (0.49) |
| 3. Standardized, evidence-based practices for management of hypertensive disorders of pregnancy | 11 | 2.73 (0.47) | 3 (0) | 3 (0) |
| 4. Screening for social determinants of health as a part of prenatal care | 10 | 2.8 (0.42) | 2.4 (0.52) | 2.78 (0.44) |
| 5. Access to midwifery/birthing center services | 10 | 2.44 (0.73) | 3 (0) | 2.9 (0.32) |
| 6. Evidence-based practices for prevention of the primary cesarean, including intermittent auscultation | 9 | 2.33 (0.71) | 2.89 (0.33) | 2.29 (0.76) |
| 7. Telehealth as a form of prenatal/postpartum care, including remote blood pressure monitoring in pregnancy and postpartum | 9 | 2.44 (0.53) | 2.78 (0.44) | 2.89 (0.33) |
| 8. Contraceptive access across the lifespan, including immediate postpartum LARC | 9 | 2.67 (0.5) | 3 (0) | 2.89 (0.33) |
| 9. Standardized, evidence-based practices for management of obstetric hemorrhage | 7 | 2.71 (0.49) | 3 (0) | 2.86 (0.38) |
| 10. Evidence-based practices for screening for and management of maternal opioid use disorder, including patient navigation services | 7 | 2.14 (0.38) | 2.43 (0.79) | 2.17 (0.41) |
| 11. Doula support | 6 | 2.17 (0.75) | 3 (0) | 3 (0) |
| 12. Implicit/racial bias training for staff | 6 | 2.5 (0.55) | 2.2 (0.45) | 2.6 (0.55) |
| 13. Maternal death reporting and review committees | 5 | 2.8 (0.45) | 2.5 (0.58) | 2.75 (0.5) |
| 14. Group prenatal care and CenteringPregnancy | 4 | 2.5 (0.58) | 3 (0) | 2.75 (0.5) |
| 15. Availability of trial of labor after cesarean | 3 | 2.67 (0.58) | 3 (0) | 2.33 (0.58) |
| 16. Appropriate use of antenatal corticosteroids in women at risk for preterm birth | 2 | 2.5 (0.71) | 2.5 (0.71) | 2 (1.4) |
| 17. Utilization of prenatal oral health care | 2 | 2 (0) | 2.5 (0.71) | 2.5 (0.71) |
| 18. Low-dose aspirin for preeclampsia prevention | 2 | 3 (0) | 2.5 (0.71) | 2 (0) |
| 19. Nutrition and lifestyle education | 2 | 1.5 (0.71) | 3 (0) | 2.5 (0.71) |
| 20. Evidence-based practices for active management of labor | 1 | 3 (0) | 3 (0) | 2 (0) |

Notes: Practices are listed in order of number of Survey #2 participants who selected them to be in the top 3 practices most recommended for implementation. Average ratings for feasibility of implementation, likelihood of improved outcomes with implementation, and likely impact on disparities are also included.**Supplemental Table 2:** Disaggregated ratings of practices most recommended for de-implementation in Survey #1, as consolidated by the investigative team.

|  | **Number of survey #2 respondents who selected this practice in top 3**  **(n=48)** | **Feasibility of de-implementing this practice in US maternity care settings**  **Scale 1-3** | **Likelihood that de-implementation of this practice will improve outcomes**  **Scale 1-3** | **Likelihood that de-implementation of this practice will reduce disparities in maternity outcomes**  **Scale 1-3** |
| --- | --- | --- | --- | --- |
| 1. Cesarean delivery for low-risk patients | 23 | 2.35 (0.59) | 2.85 (0.37) | 2.65 (0.61) |
| 2. Routinely discontinuing all psychiatric medications during pregnancy, without medical indication for doing so | 22 | 2.65 (0.59) | 2.8 (0.41) | 2.29 (0.73) |
| 3. Routine separation of infants and parents at birth | 14 | 2.69 (0.48) | 2.92 (0.29) | 2.6 (0.70) |
| 4. Routine continuous electronic fetal monitoring | 12 | 1.83 (0.72) | 2.55 (0.52) | 1.88 (0.64) |
| 5. Routine induction without medical indication | 10 | 1.8 (0.63) | 2.6 (0.52) | 2.22 (0.67) |
| 6. Unindicated urine drug screening during perinatal care | 10 | 2.55 (0.53) | 2.67 (0.5) | 2.78 (0.44) |
| 7. Excessive opioid prescribing post-cesarean | 10 | 2.6 (0.52) | 3 (0) | 2.25 (0.71) |
| 8. Standard 12-14 prenatal visit schedule for low-risk people | 6 | 2.67 (0.52) | 2 (0.63) | 2.4 (0.55) |
| 9. Reduced movement in labor | 6 | 2.6 (0.55) | 3 (0) | 2.5 (0.58) |
| 10. Oral intake restrictions during labor | 5 | 2.4 (0.55) | 2 (0) | 1.67 (0.58) |
| 11. Bedrest for antenatal conditions | 4 | 2.75 (0.5) | 2.75 (0.5) | 2 (1) |
| 12. Unindicated ultrasounds | 3 | 1.67 (0.58) | 1.67 (0.58) | 2 (0) |
| 13. Maternal oxygen supplementation during labor | 2 | 2.5 (0.71) | 3 (0) | 1 (0) |
| 14. Overuse of vital signs in labor | 2 | 2.5 (0.71) | 3 (0) | 2.5 (0.71) |
| 15. Routine amniotomy | 1 | 3 (0) | 3 (0) | 2 (0) |
| 16. Early screening for gestational diabetes | 1 | 3 (0) | 3 (0) | 3 (0) |

Notes: Practices are listed in order of number of Survey #2 participants who selected them to be in the top 3 practices most recommended for de-implementation. Average ratings for feasibility of implementation, likelihood of improved outcomes with implementation, and likely impact on disparities are also included.
